# Supplementary material for: Water cluster in hydrophobic crystalline porous covalent organic frameworks
Source: Nat Commun. 2021 Nov 19;12:6747. doi: 10.1038/s41467-021-27128-4 (PMC8604923; doi:10.1038/s41467-021-27128-4)
Supplement: Supplementary file 1 — Supplementary Information [file 41467_2021_27128_MOESM1_ESM.pdf]

Supplementary Information

Water cluster in hydrophobic crystalline porous covalent organic frameworks

Ke Tian Tan, Shanshan Tao, Ning Huang & Donglin Jiang★

Department of Chemistry, Faculty of Science, National University of Singapore, 3, Science Drive 3, Singapore 117543, Singapore.

★Corresponding author: Prof. Donglin Jiang (chmjd@nus.edu.sg)

Table of content

Supplementary tables 1 – 4 -- S2

Supplementary figures 1 – 13 --- S7

Supplementary Table 1 | Atomistic coordinates for the AA-stacking mode of HFPTP-DMePDA-COF; space group *P3*, *a* = *b* =25.8092 Å, *c* = 4.5279 Å, *α* = *β* = 90° and *γ* = 120°.

Atom number	atom	*x*	*y*	*z*
C1	C	-1.27748	-0.61239	0.32928
C2	C	-1.22273	-0.5575	0.32685
C3	C	-1.21898	-0.50246	0.39035
C4	C	-1.27326	-0.50113	0.4132
C5	C	-0.3315	-0.61069	0.33747
C6	C	-0.32787	-0.55458	0.37964
H7	H	-0.37045	-0.5516	0.38741
H8	H	-0.44323	-0.6225	0.27173
C9	C	-1.2762	-0.44652	0.48971
C10	C	-1.24204	-0.40998	0.72202
C11	C	-0.31467	-0.43178	2.33797
C12	C	-1.24089	-0.35646	0.7843
H13	H	-1.21446	-0.42364	0.86468
C14	C	-0.31529	-0.37916	2.40545
H15	H	-0.34548	-0.46306	2.15891
C16	C	-1.27739	-0.34044	0.62605
H17	H	-1.21015	-0.32557	0.96468
H18	H	-0.3466	-0.36759	2.28133
C19	C	-1.27412	-0.28288	0.69134
N20	N	-0.30259	-0.2636	2.52978
H21	H	-1.24632	-0.25462	0.88591
C22	C	-0.29936	-0.2065	2.55871
C23	C	-0.34381	-0.19806	2.42555
C24	C	-1.25079	-0.15762	0.69929
C25	C	-0.34015	-0.14211	2.43672
C26	C	-0.39488	-0.24816	2.26561
C27	C	-1.24681	-0.10156	0.70693
H28	H	-1.21454	-0.1641	0.80741
C29	C	-0.29163	-0.09351	2.57691
H30	H	-0.37671	-0.13573	2.33158
C31	C	-1.19415	-0.05049	0.85298
N32	N	-0.28414	-0.03461	2.56368
C33	C	-0.32725	-0.02257	0.57779
H34	H	-0.37503	-0.06002	0.62015
C35	C	-0.31452	0.03941	0.53913
C36	C	-0.35462	0.05656	0.64582
C37	C	-1.26286	0.08161	2.39069
C38	C	-0.34334	0.11498	0.60309
H39	H	-0.39672	0.02285	0.76826

C40	C	-1.25216	0.13953	2.34379
H41	H	-1.22945	0.06825	2.30804
C42	C	-1.2924	0.1574	0.44563
H43	H	-0.37552	0.12903	0.69597
H44	H	-1.21021	0.17343	2.221
H45	H	-0.39377	-0.2913	2.30191
H46	H	-0.39104	-0.23758	2.01945
H47	H	-0.43884	-0.25386	2.35334
H48	H	-1.1656	-0.01585	0.6796
H49	H	-1.16631	-0.06754	0.96839
H50	H	-1.21035	-0.02908	1.02242

Supplementary Table 2 | Porosity of COFs

COF	BET surface area (m^2 g^{-1})	Pore size (nm)	Pore volume (cm^3 g^{-1})
**HFPTP-PDA-COF**	662	1.1, 1.4	0.32
**HFPTP-DMePDA-COF**	480	1.1, 1.4	0.24
**HFPTP-BPDA-COF**	758	1.2, 1.6	0.39
**TFBCz-PDA-COF**	1221	1.5	0.60
**TFPPy-PDA-COF**	1532	2.1	0.76
**TTA-TFB-COF**	1244	1.5	0.55
**TPB-DMTP-COF**	2346	3.2	1.08

Supplementary Table 3 | Vapour sorption of COFs

COF	$\alpha^{\#}$ (P/P_0)			Uptake capacity at 0.9 P/P_0 (g g^{-1})			Pore Occupancy at 0.9 P/P_0 (%)			Q_{st} (kJ mol^{-1})
	10 °C	15 °C	25 °C	10 °C	15°C	25 °C	10 °C	15°C	25 °C	
**HFPTP-PDA-COF**	0.42	0.42	0.42	0.27	0.29	0.30	84	91	93	44.8
**HFPTP-DMePDA-COF**	0.80	0.80	0.80	0.10	0.15	0.12	43	61	49	43.5
**HFPTP-BPDA-COF**	0.52	0.52	0.55	0.32	0.34	0.35	81	86	90	43.7
**TFBCz-PDA-COF**	0.42	0.42	0.42	0.48	0.49	0.47	79	81	79	46.7
**TFPPy-PDA-COF**	0.58	0.58	0.58	0.62	0.57	0.65	81	75	85	41.4
**TTA-TFB-COF**	0.45	0.45	0.45	0.51	0.51	0.53	92	93	97	45.6
**TPB-DMTP-COF**	0.77	0.77	0.77	0.64	0.65	0.71	59	60	66	43.3

α – Relative pressure at which the uptake amount is half of that at 0.9 P/P_0.

Supplementary Table 4 | Elemental analysis of COFs

COF		C%	H%	N%
HFPTP-PDA-COF	Calculated	87.62	4.52	7.86
	Observed	81.45	4.66	7.54
HFPTP-DMePDA-COF	Calculated	87.47	5.24	7.29
	Observed	84.46	4.91	6.05
HFPTP-BPDA-COF	Calculated	88.86	4.66	6.48
	Observed	84.88	4.60	5.96
TFBCz-PDA-COF	Calculated	81.61	4.11	14.28
	Observed	76.83	3.96	13.40
TFPPy-PDA-COF	Calculated	87.47	5.24	7.29
	Observed	84.03	4.30	6.91
TTA-TFB-COF	Calculated	77.91	3.92	18.17
	Observed	73.39	3.77	16.88
TPB-DMTP-COF	Calculated	79.57	5.14	7.14
	Observed	76.46	4.84	7.36

^{13}C CP-MAS NMR Spectrum

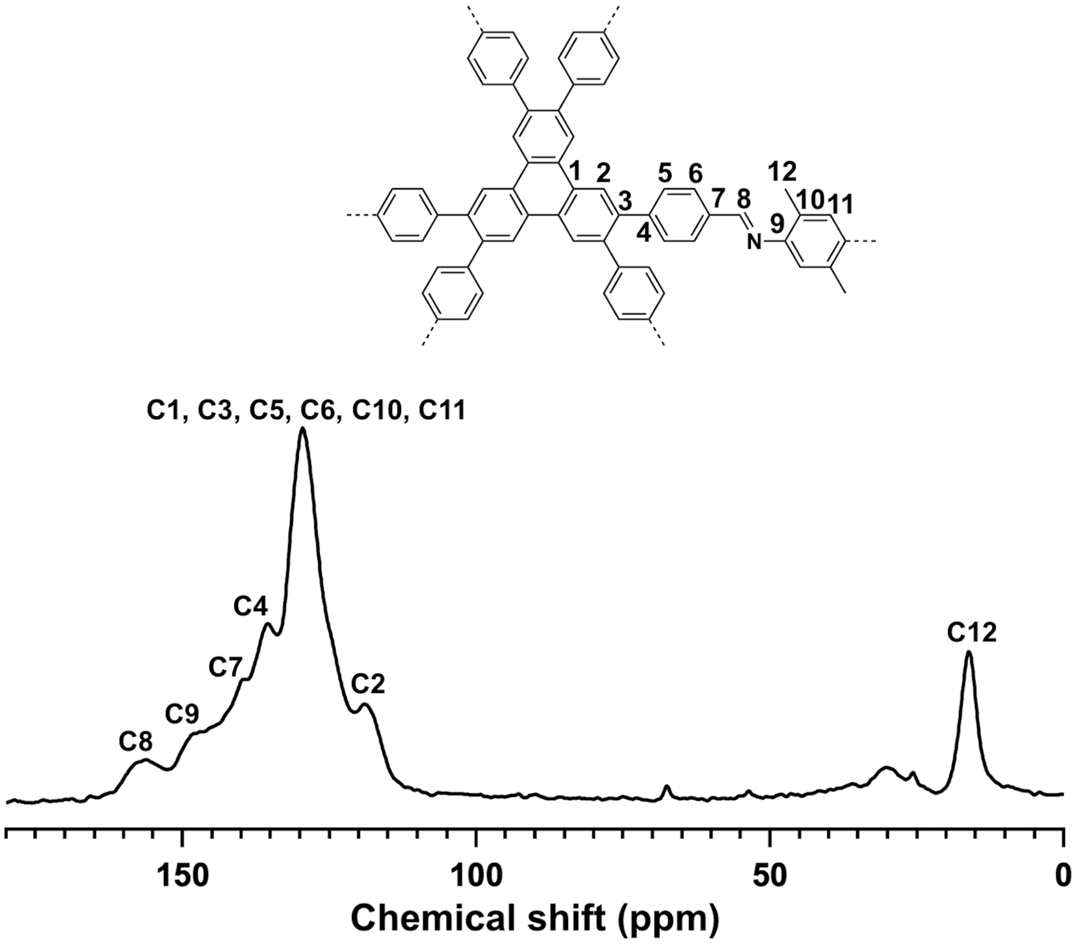

Supplementary Fig. 1 | Solid-state ^{13}C CP/MAS NMR spectrum of HFPTP-DMePDA-COF. Signal at 156 ppm (C8) was assigned to the C atom of the C=N imine linkage.

FT IR Spectra

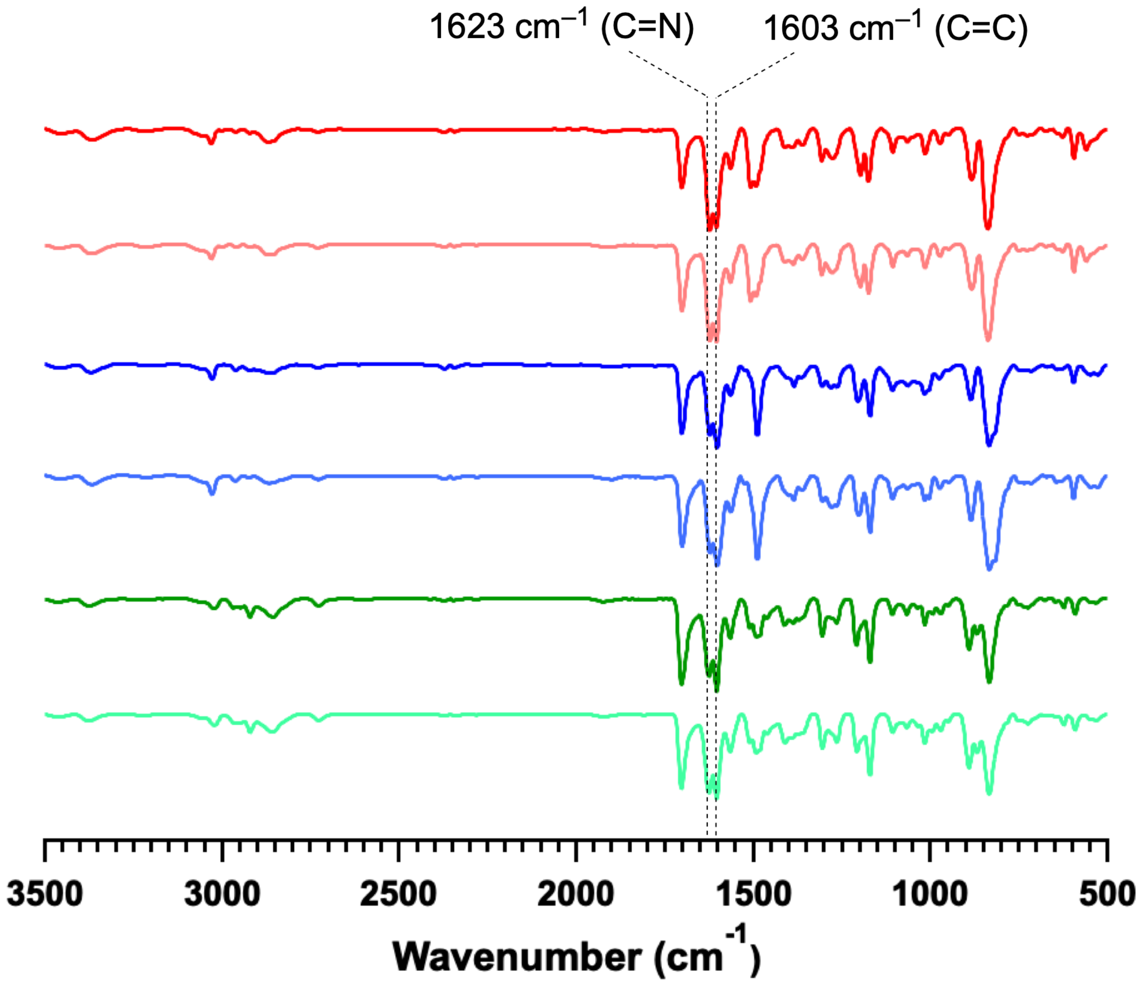

Supplementary Fig. 2 | FT IR spectra. Red line – HFPTP-PDA-COF before vapour sorption, light red line -HFPTP-PDA-COF after vapour sorption, blue line – HFPTP-DMePDA-COF before vapour sorption, light blue line – HFPTP-DMePDA-COF after vapour sorption, green line – HFPTP-BPDA-COF before vapour sorption and light green line – HFPTP-BPDA-COF after vapour sorption.

Characterization of TFBCz-PDA-COF and TTA-TFB-COF

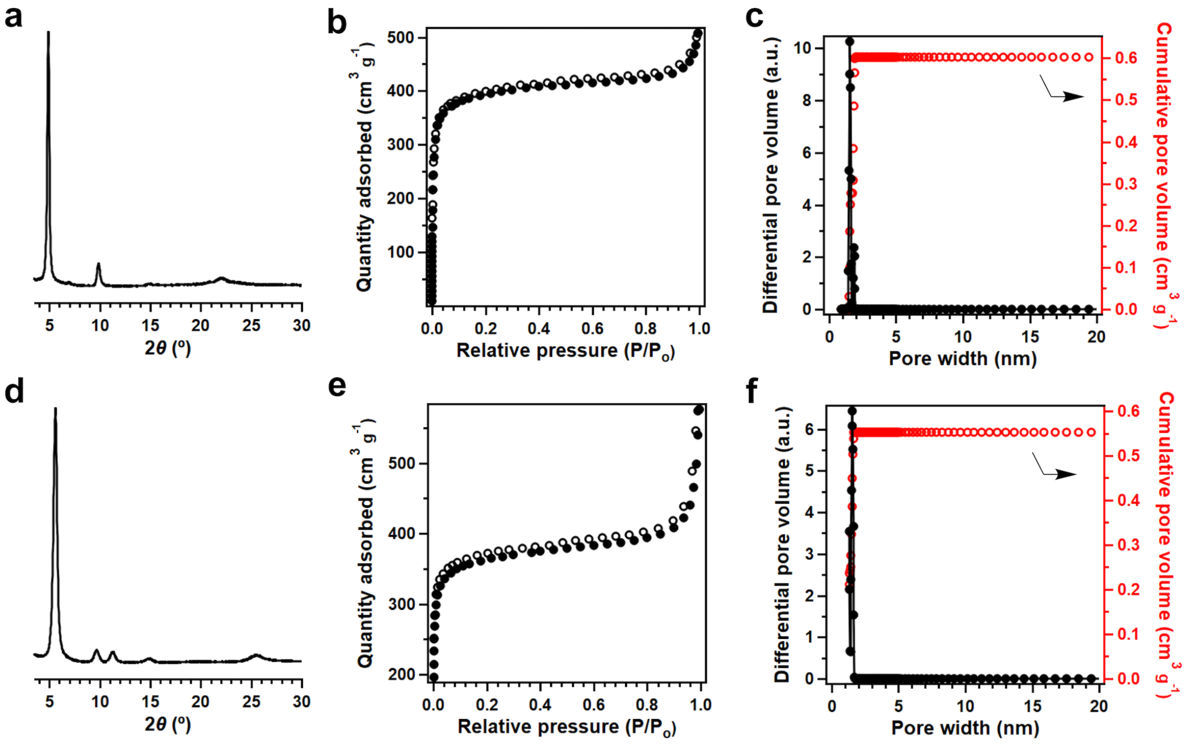

Supplementary Fig. 3 | PXRD and porosity. a–c, (a) PXRD pattern, (b) N_2 sorption isotherm and (c) pore size distribution and pore volume of TFBCz-PDA-COF. **d–f**, (d) PXRD pattern, (e) N_2 sorption isotherm and (f) pore size distribution and pore volume of TTA-TFB-COF.

Characterization of TFPPy-PDA-COF and TPB-DMTP-COF

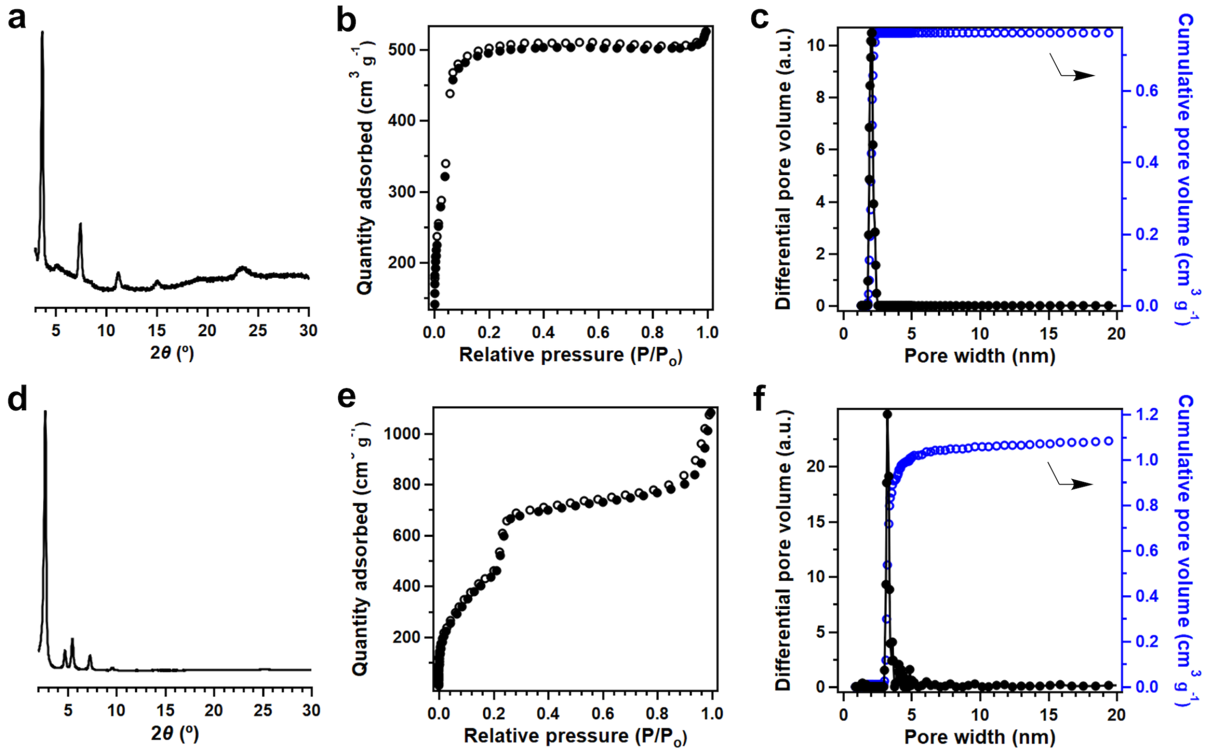

Supplementary Fig. 4 | PXRD and porosity of tetragonal and hexagonal mesoporous COFs. **a–c**, (a) PXRD pattern, (b) N_2 sorption isotherm and (c) pore size distribution and pore volume of TFPPy-PDA-COF. **d–f**, (d) PXRD pattern, (e) N_2 sorption isotherm and (f) pore size distribution and pore volume of TPB-DMTP-COF.

Cycling performance of microporous COFs

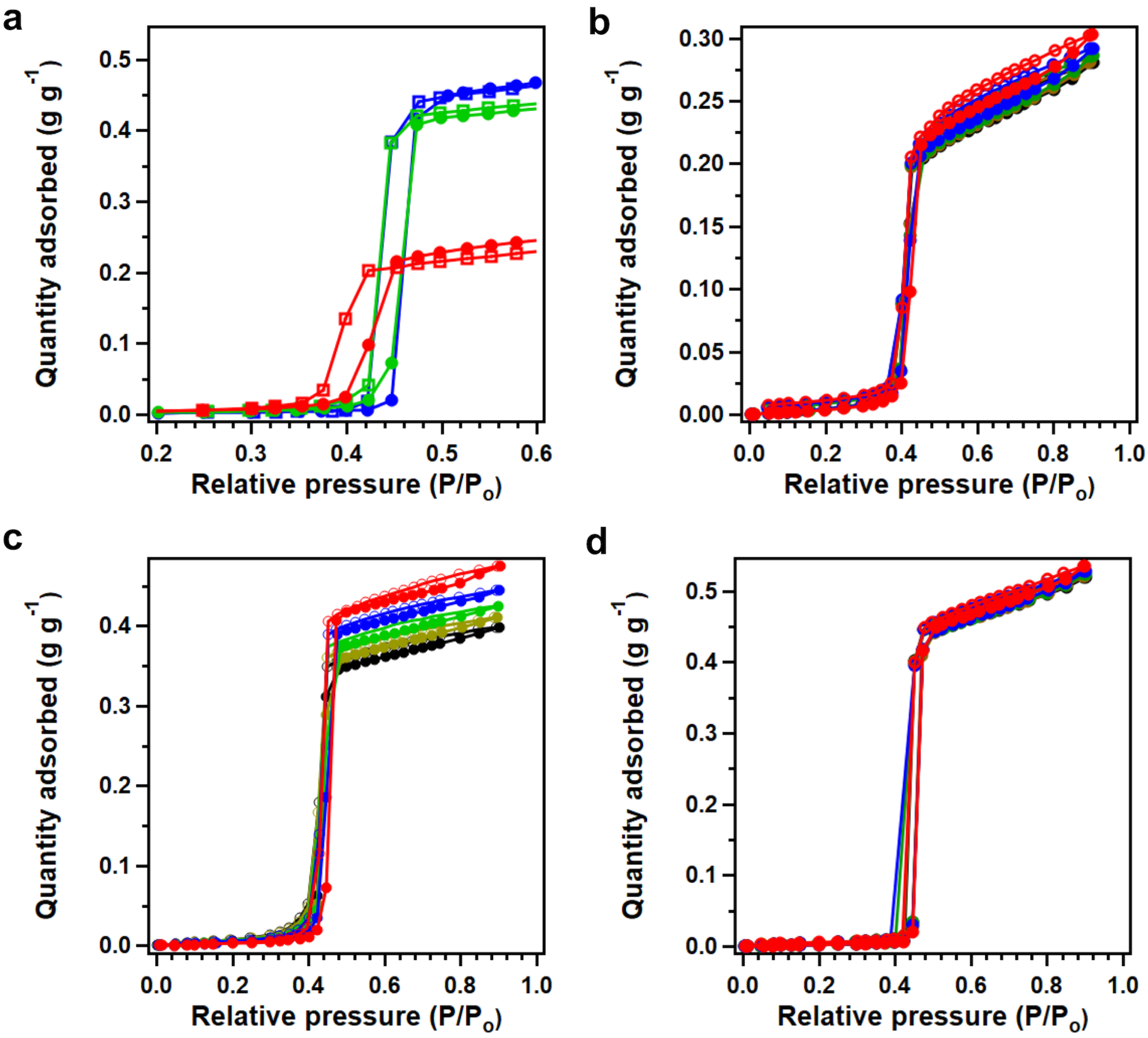

Supplementary Fig. 5 | Water vapour adsorption and cycle performance. a, Adsorption branch of HFPTP-PDA-COF (red), TFBCz-PDA-COF (green) and TTA-TFB-COF (blue) measured at 25 °C (filled circle) and 10 °C (empty rectangle). **b–d**, Five consecutive water sorption isotherms of (b) HFPTP-PDA-COF, (c) TFBCz-PDA-COF and (d) TTA-TFB-COF (line with filled dots – adsorption branch, line with empty dots – desorption branch, red – first cycle, blue – second cycle, green – third cycle, dark yellow – fourth cycle, black – fifth cycle).

Characterization of TTA-TFB-CMP

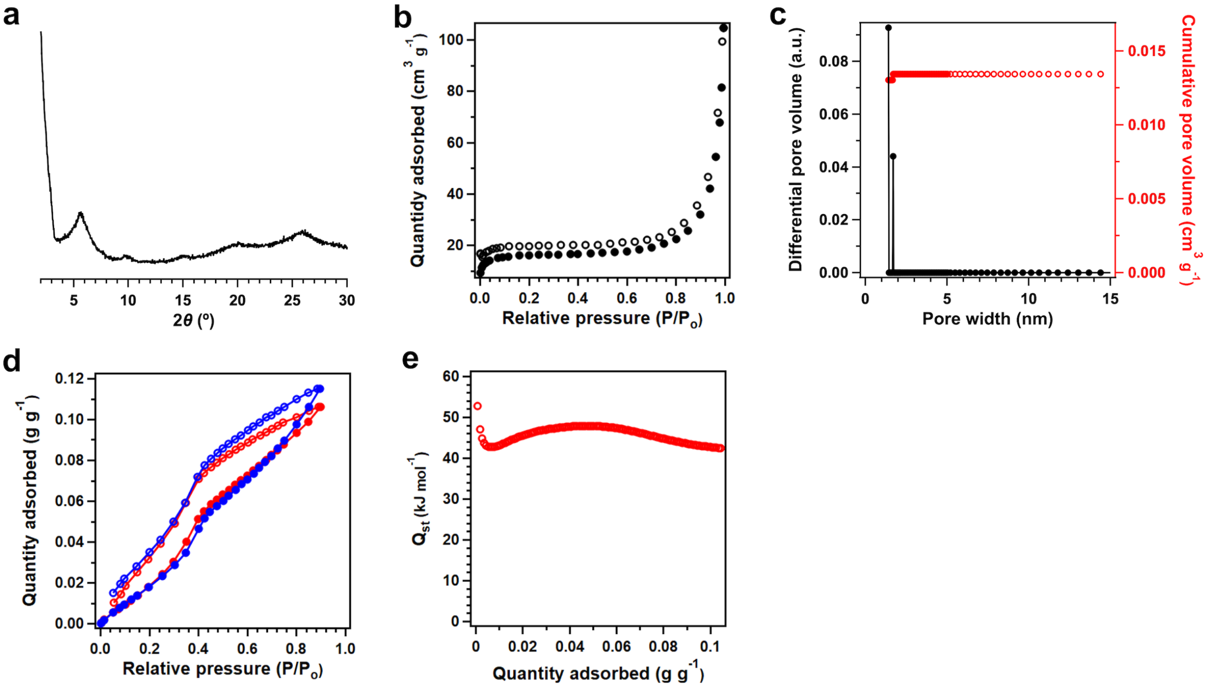

Supplementary Fig. 6 | Low crystalline TTA-TFB-CMP. a–e, (a) PXRD pattern, (b) nitrogen sorption isotherm, (c) pore size distribution and pore volume, (d) vapour sorption isotherm measured at 25 °C (blue) and 10 °C (red) (Filled circle – adsorption branch, empty circle – desorption branch) and (e) Q_{st} plot of TTA-TFB-CMP.

Isosteric heat of adsorption (Q_{st}) plots

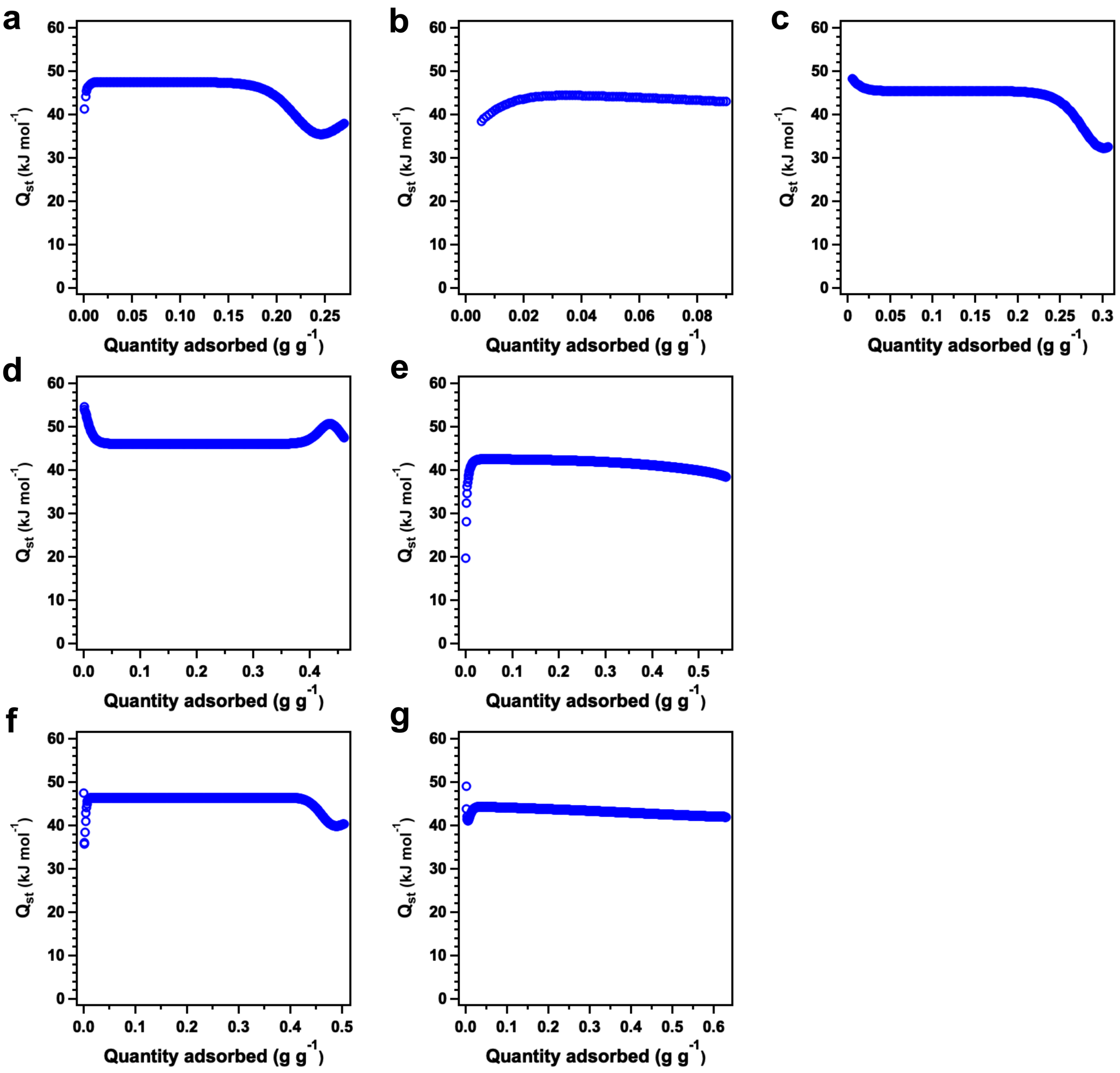

Supplementary Fig. 7 | Isosteric heat of trigonal, tetragonal and hexagonal microporous and mesoporous COFs. a–g, Q_{st} plots of (a) trigonal microporous HFPTP-PDA-COF, (b) trigonal microporous HFPTP-DMePDA-COF, (c) trigonal microporous HFPTP-BPDA-COF, (d) tetragonal microporous TFBCz-PDA-COF, (e) tetragonal mesoporous TFPPy-PDA-COF, (f) hexagonal microporous TTA-TFB-COF, and (g) hexagonal mesoporous TPB-DMTP-COF.

PXRD patterns after water adsorption

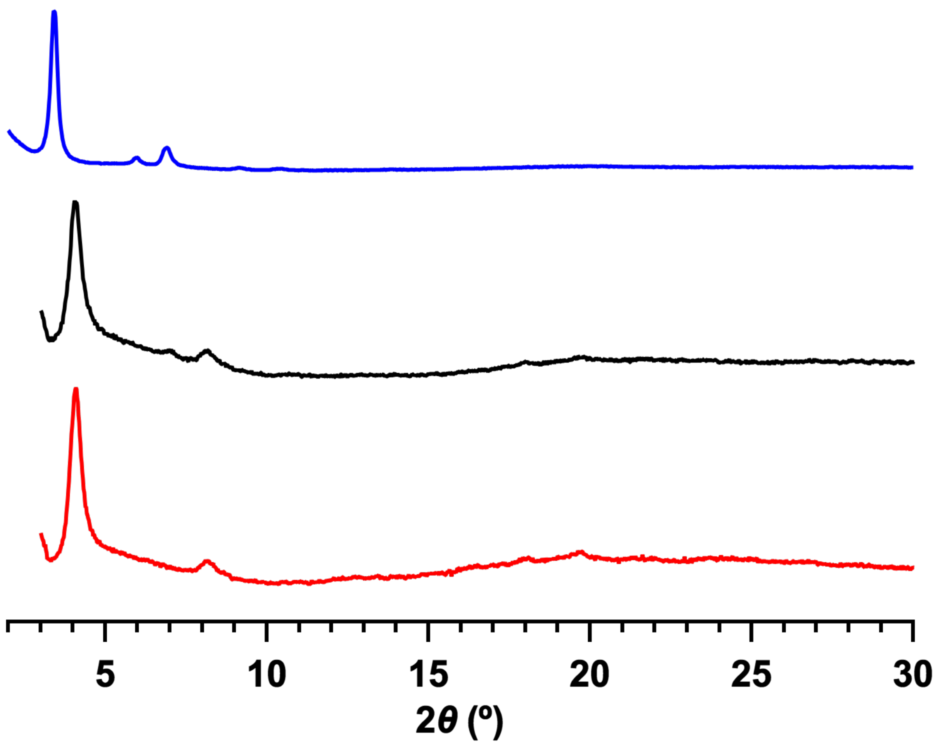

Supplementary Fig. 8 | PXRD patterns of trigonal COFs after vapour sorption. Red line – HFPTP-PDA-COF, black line – HFPTP-DMePDA-COF and blue line – HFPTP-BPDA-COF.

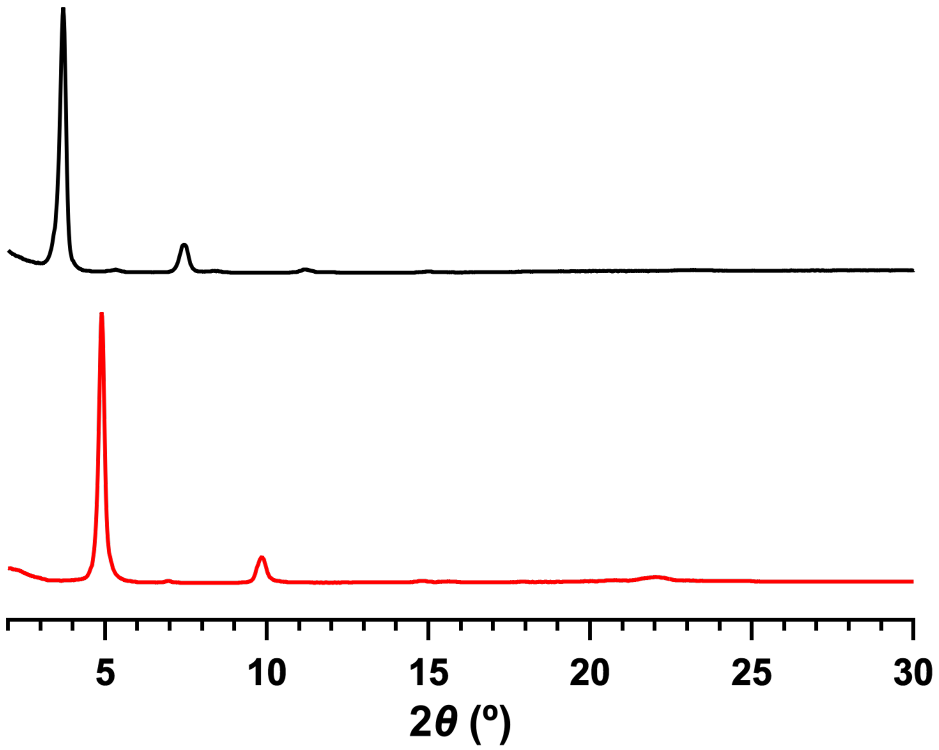

Supplementary Fig. 9 | PXRD patterns of tetragonal COFs after vapour sorption. Red line – TFBCz-PDA-COF and black line – TFPPy-PDA-COF.

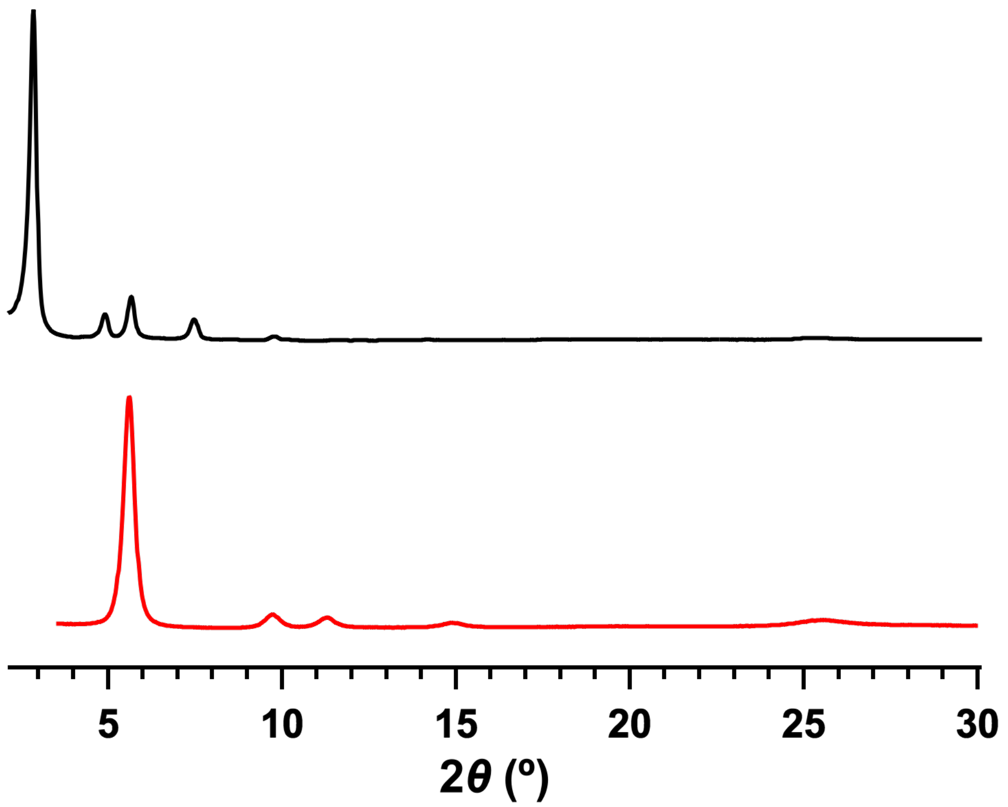

Supplementary Fig. 10 | PXRD patterns of hexagonal COFs after vapour sorption. Red line – TTA-TFB-COF and black line – TPB-DMTP-COF.

FT IR Spectra

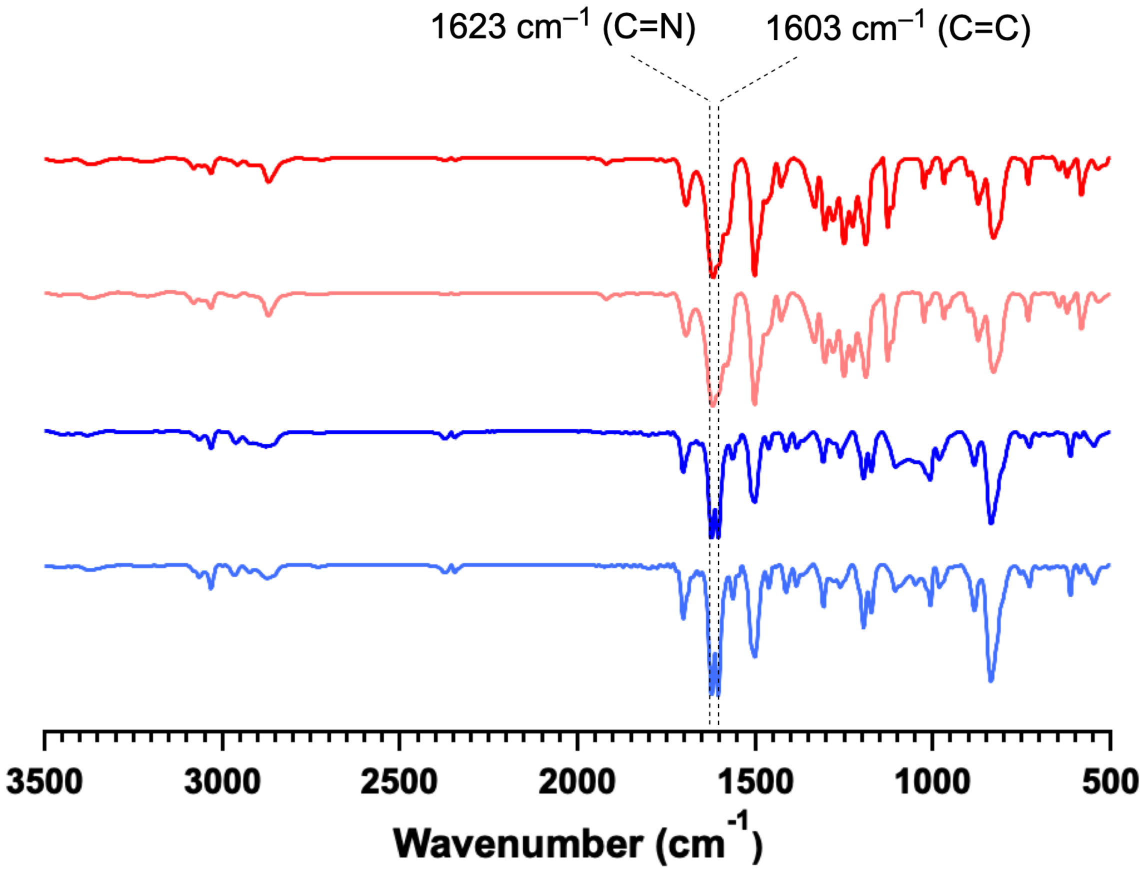

Supplementary Fig. 11 | FT IR spectra. Red line – TFBCz-PDA-COF before vapour sorption, light red line -TFBCz-PDA-COF after vapour sorption, blue line – TFPPy-PDA-COF before vapour sorption and light blue line – TFPPy-PDA-COF after vapour sorption.

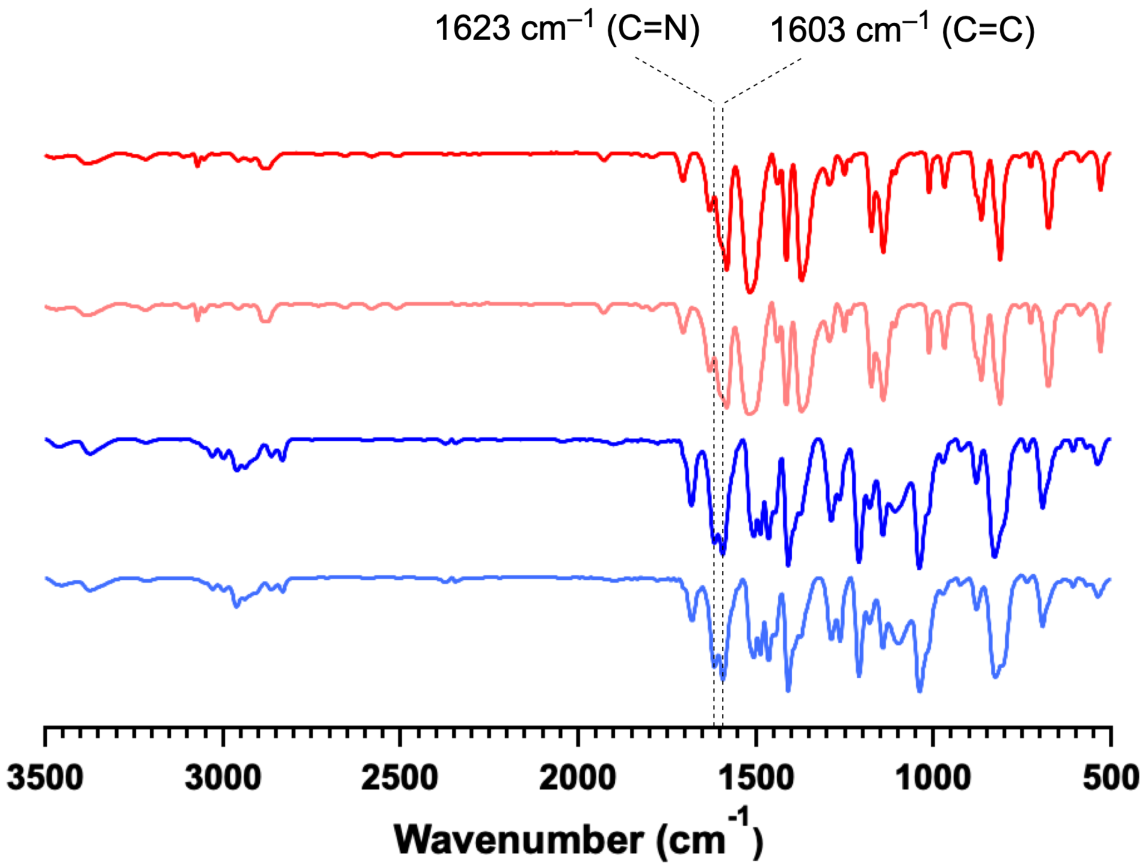

Supplementary Fig. 12 | FT IR spectra. Red line – TTA-TFB-COF before vapour sorption, light red line -TTA-TFB-COF after vapour sorption, blue line – TPB-DMTP-COF before vapour sorption and light blue line – TPB-DMTP-COF after vapour sorption.

Porosity of HFPTP-PDA-COF after vapour sorption

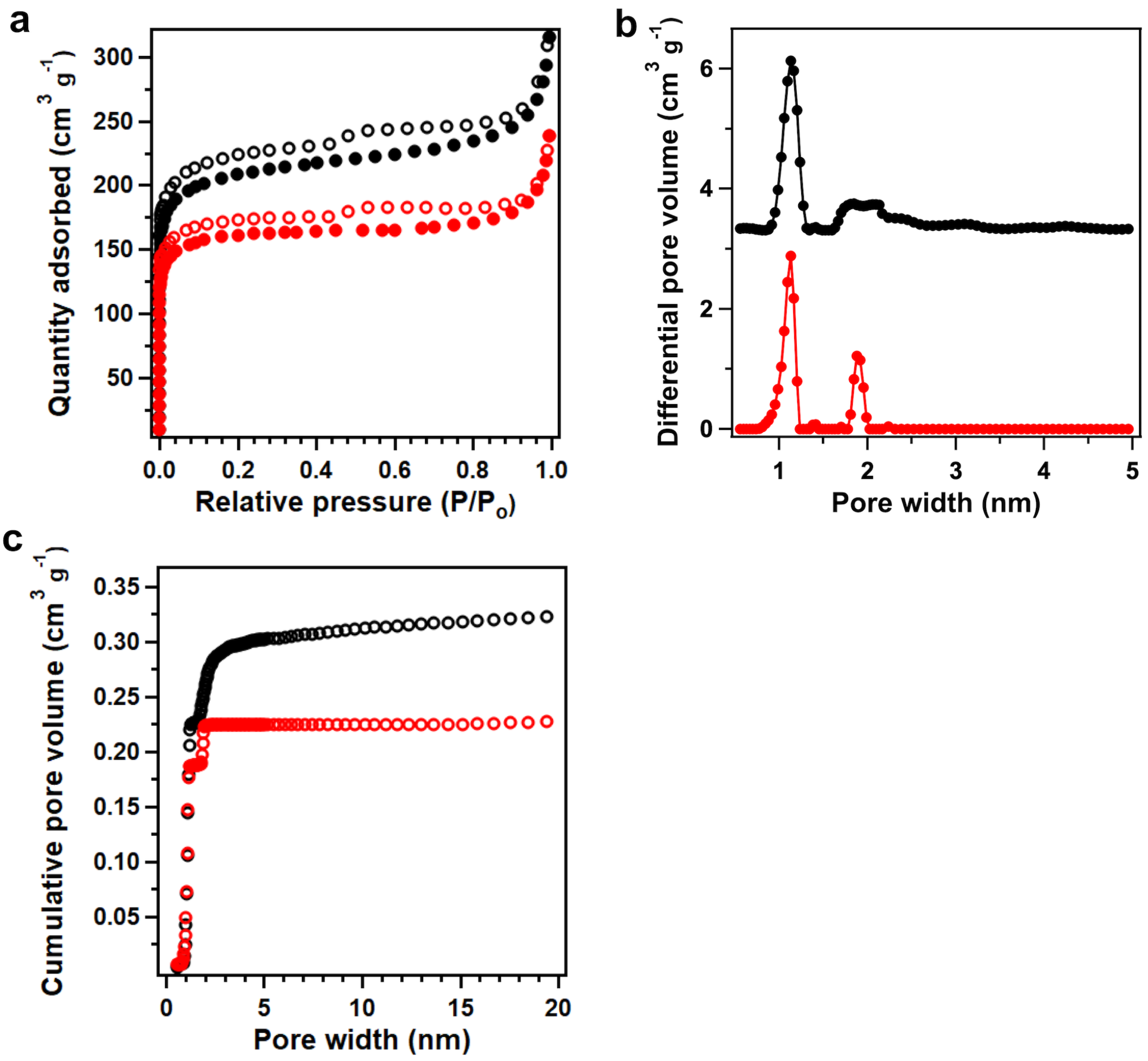

Supplementary Fig. 13 | Porosity after cycle use. **a–c**, (a) N_2 sorption isotherm, (b) pore size distribution and (c) pore volume of HFPTP-PDA-COF (black line – before vapour sorption, red line – after 5 cycles of vapour sorption).
